# Evaluation of three scoring systems for predicting renal prognosis in antineutrophil cytoplasmic antibody-associated glomerulonephritis

**DOI:** 10.1186/s40001-023-01113-7

**Published:** 2023-05-09

**Authors:** Ruiqiang Wang, Xiaofeng Zhang, Xinfang Wang, Lin Chen, Qiuling Ma, Yajing Su, Jingwen Liu, Huihui Shi

**Affiliations:** 1 Department of Nephrology, The First Affiliated Hospital of Zhengzhou University, No.1 Jianshe Road, Zhengzhou, 450052 Henan China; 2 Department of Hematology, The Second Affiliated Hospital of Henan University of Traditional Chinese Medicine, No.6 Dongfeng Road, Zhengzhou, 450046 Henan China; 3 Institute of Hematology, Henan University of Chinese Medicine, Zhengzhou, 450002 Henan China

**Keywords:** Antineutrophil cytoplasmic antibody-associated glomerulonephritis, Renal risk score, Renal vascular lesions score, Birmingham vasculitis activity score, Renal prognosis

## Abstract

**Background:**

Antineutrophil cytoplasmic antibody (ANCA)-associated glomerulonephritis (AAGN) is characterized by rapidly progressive glomerulonephritis, and timely initiation of treatment and evaluation is critical to prevent the progression of renal disease to end-stage renal disease (ESRD). The aim of this study was to evaluate predictive value of the renal risk score (RRS), Birmingham vasculitis activity score (BVAS), and renal vascular lesions (RVLs) score for renal prognosis in AAGN.

**Methods:**

A retrospective analysis of ninety-four patients diagnosed with AAGN after renal biopsy was performed. The RRS, BVAS, and RVLs score were evaluated in relation to clinicopathologic features and renal prognosis. A receiver operating characteristic curve (ROC) was used to evaluate their renal prognostic value.

**Results:**

The median follow-up time was 36 months. Thirty-eight patients progressed to ESRD. Survival analysis showed that renal prognosis worsened in the RRS group in order of low, medium, and high RRS (*P* < 0.05). Within the RVLs group, the renal prognosis of the groups with severe and moderate RVLs was worse than that of the group without RVLs (*P* = 0.012, *P* < 0.001), and the group with mild RVLs was close to that of the group without RVLs. ROC analysis showed that the AUC of RRS, BVAS, RVLs score, RVLs score combined with RRS (RVLs score & RRS, RR), RVLs score, and RRS combined with BVAS (RVLs score & RRS & BVAS, RRB) were 0.865, 0.624, 0.763, 0.910, and 0.942, respectively. The predictive power of RRB and RR was comparable and significantly better than the RRS, BVAS, and RVLs score. Based on simplicity and validity, RR was selected as the best predictor, and the relationship between RRS, RVLs score, and RR was calculated using a linear fit, resulting in the linear equation RR = -0.4766 + 0.1231 × RVLs score + 0.395 × RRS (*P* < 0.001).

**Conclusions:**

In AAGN, the predictive power of RR for renal prognosis was superior to that of RRS, BVAS, and RVLs score. RR may serve as a new predictor of renal prognosis in AAGN.

## Background

The nomenclature of the primary systemic vasculitis syndromes was defined by the 1994 Chapel Hill Consensus Conference (CHCC). The CHCC definitions were based on the predominant size of blood vessel involvement in tissue biopsies (large, medium, and small vasculitis). In the 2012 CHCC system, small-vessel vasculitis was subdivided into the immune complex small-vessel vasculitis and antineutrophil cytoplasmic antibody (ANCA)-associated vasculitis (AAV) [1]. AAV has been officially defined as a group of pauci-immune small-vessel vasculitides closely related to ANCA and specific for myeloperoxidase (MPO) or proteinase 3 (PR3), with inflammation and necrosis of the small vessel walls as the main manifestations. Phenotypes include granulomatosis with polyangiitis, microscopic polyangiitis, and eosinophilic granulomatosis with polyangiitis [2]. The renal is one of the most commonly involved organs in AAV, with more than 75% of patients having renal involvement [3]. Under immunofluorescence microscope, it showed pauci-immune pattern; Under the light microscope, it showed necrotic and crescentic glomerulonephritis, which is characterized by inflammation of small vessels, fibrinoid necrosis of the collateral branches of glomerular capillaries and extracapillary hyperplasia. In addition to glomerular lesions, renal tubular injury, interstitial mononuclear cell infiltration and fibrosis were also observed; The swelling of endothelial cells, formation of microthrombosis and degranulation of neutrophils can be observed under the electron microscope, but there is no deposition of electronic dense matter. Clinical features presented as rapidly progressive glomerulonephritis with decreased renal function, oliguria or anuria, or may be accompanied by proteinuria, microscopic haematuria and hypertension. Renal involvement of AAV is often associated with poor renal and patient outcomes. Despite intensive treatment, a significant percentage of patients achieve end-stage renal disease (ESRD) [1].

The renal risk score (RRS) is based on three parameters-estimated glomerular filtration rate (eGFR), percentage of normal glomeruli, and rate of renal tubular atrophy and interstitial fibrosis (TA /IF)—which are classified into three groups according to severity. There is increasing evidence that RRS plays an important role in predicting renal prognosis in ANCA-associated glomerulonephritis (AAGN) [4–6]. Chen et al. reported that Birmingham Vasculitis Activity Score (BVAS) was associated with poor renal prognosis in AAGN [7]. In addition, our previous study found that renal vascular lesions (RVLs) (extraglomerular vascular lesions, including arterial fibrotic intimal thickening and arteriolohyalinosis) affected the renal prognosis of AAGN [8]. However, few studies have investigated the prognostic significance of RRS, BVAS, and RVLs score for the kidneys. This study focused on analysing their relationship with renal prognosis and comparing their predictive power.

## Materials and methods

### Patients

Ninety-four patients who underwent renal biopsy and were diagnosed with AAGN between April 2014 and May 2021 at First Affiliated Hospital of Zhengzhou University were enrolled in this study. Inclusion criteria: (1) a positive ANCA detection by indirect immunofluorescence, a positive MPO detection, or a positive PR3 detection by antigen-specific enzyme-linked immunosorbent assay; (2) meeting the CHCC criteria for defining AAV; (3) performance of a renal biopsy and detection of ≥ 8 glomeruli in the renal biopsy specimen; (4) at least 12 months of follow-up (including patients who died within 12 months but excluding patients lost to follow-up). Exclusion criteria: Patients with concurrent glomerular disease, such as IgA nephropathy, membranous nephropathy, anti-glomerular basement membrane, or lupus nephritis. This study was approved by the Ethics Committee of First Affiliated Hospital of Zhengzhou University (Ethics Review Number: 2019-KY-015).

### Data collection

Clinical and laboratory data were collected during renal biopsy: Age, sex, systolic blood pressure (SBP), diastolic blood pressure (DBP), hemoglobin (HB), platelet count, albumin (ALB), serum creatinine (SCr), uric acid (UA), eGFR (calculated according to the Chronic Kidney Disease Epidemiology Collaboration equation), C-reactive protein (CRP), complement C3, complement C4, urinary red blood cell count, urinary protein, BVAS (3 Version).

### Histopathology of the kidneys

Histopathology included approximate normal glomerular proportion, global glomerulosclerosis proportion, cellular crescent proportion, fibrocellular crescent proportion, fibrous crescent proportion, TA/IF, interstitial inflammatory cell infiltration score, and microangiopathy. The renal biopsy specimens were examined by light microscopy, electron microscopy, and immunofluorescence. These specimens were reviewed and scored by two independent and experienced pathologists who were unaware of the patient's clinical indicators.

The semi-quantitative scoring system was used for scoring and grouping. RRS was scored according to the percentage of normal glomeruli (N0 > 25%, N1 10% to 25%, N2 < 10%), the percentage TA/IF (T0 ≤ 25%, T1 > 25%), and eGFR at diagnosis (G0 > 15 ml/min/1.73 m^2^, G 1 ≤ 15 ml/min/1.73 m^2^) (N1 = 4, N2 = 6, T1 = 2, G1 = 3), and divided into three groups: 0 = low RRS group; 2–7 = medium RRS group; and 8–11 = high RRS group.

RVLs score was determined by intima-media thickness (normal 0, ≤ media thickness 1, > media thickness 2) and vitreous changes (absent 0, present 1) and divided into four groups: 0 = group with no RVLs; 1 = group with mild RVLs; 2 = group with moderate RVLs; and 3 = group with severe RVLs.

### Treatment regimen

Induction of remission with glucocorticoids and/or combined immunosuppressants. Prednisone 1 mg/kg/d for 4–6 weeks with gradual reduction after disease control; combined with cyclophosphamide (CTX) 0.5–1.0 g/m^2^ intravenous drip once a month or 1.5–2.0 mg/kg/day orally; combined with mycophenolate mofetil (MMF) 0.50–0.75 g twice daily in some patients. Severe cases, such as severe pulmonary hemorrhage or renal pathology manifested as crescentic nephritis and fibrinoid necrosis of small vessels, received high-dose glucocorticoids (0.25–0.50 g/d × 3 days). Maintenance remission was treated with low-dose glucocorticoids or in combination with MMF 0.25–0.50 g twice daily. Some patients are treated with biologic agents (rituximab). Severely ill patients are treated with hemodialysis or plasma exchange, intravenous infusion of gamma globulin, etc.

### Renal outcomes

The primary endpoint was progression to ESRD over time (censored for death). ESRD was defined as the need for long-term renal replacement therapy or renal transplantation. The time from baseline at renal biopsy to the last follow-up or until ESRD was achieved was calculated. Remission was defined as the absence of active disease (BVAS = 0) for at least 3 months with a prednisone dose or equivalent ≤ 7.5 mg/day.

### Statistical analysis

Categorical variables were summarized as frequencies (*n*) with percentages (%). Continuous variables were presented as mean ± standard deviation or median (25–75% interquartile range). Differences between groups were analyzed by the one-way ANOVA, Kruskal–Wallis test, and chi-square test. Cumulative renal survival was measured by the Kaplan–Meier method, and differences were compared by the Log-rank test. Univariate and multivariate Cox regression analyzes were performed to determine the predictive factors. By combining Cox regression results and clinical reality, the base model was developed. The discriminatory power to predict dialysis dependence was assessed by the area under the subject operating characteristic curve (AUC). We used the C statistic to compare the differences between the models and calculated their net reclassification index (NRI) and integrated discriminant index (IDI). *P* < 0.05 was considered statistically significant. The R language and MedCalc were used for all statistical analysis.

## Results

### Patients

**Fig. 1:**
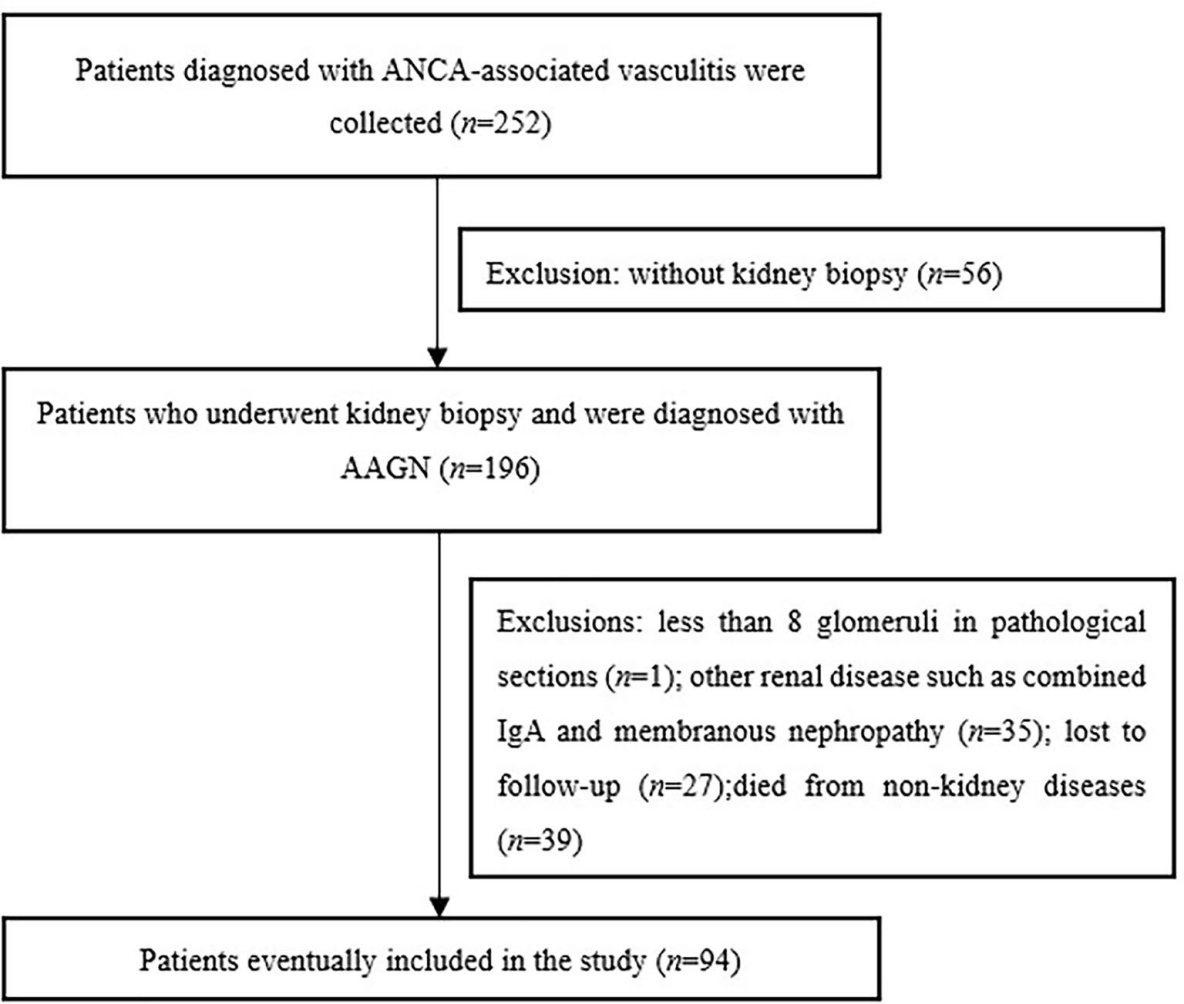
Flow diagram depicting the patients’ selection process

According to the inclusion criteria, a total of ninety-four patients were enrolled in this study(Fig. 1). Forty-six were male and forty-eight were female with an age of 60.0 (49.0, 67.0) years at renal biopsy. The MPO-ANCA was positive in 87.2% of patients and PR3-ANCA in 12.8% of patients. The median eGFR was 17.5 (9.3, 40.1) ml/min/1.73 m^2^ and seventeen (18.1%) patients required dialysis for initial treatment (Table 1).

**Table 1:** Clinical baseline characteristics

Characteristics	Total (*n* = 94)
Age (year)	60.0 (49.0, 67.0)
Female, *n* (%)	48.0 (51.1)
PR3-ANCA, *n* (%)	12.0 (12.8)
MPO-ANCA, *n* (%)	82.0 (87.2)
eGFR (ml/min/1.73 m^2^)	17.5 (9.3, 40.1)
Urinary protein (g/d)	1.8 (1.0, 2.7)
Urinary red blood cells (cells/μl)	148.5 (54.0, 286.8)
Normal glomeruli (%)	32.5 (12.8, 59.7)
TA/IF (> 25%), *n* (%)	17.0 (18.1)
RRS	3.0 (0, 7.0)
BVAS	18.0 (15.0, 21.0)
RVLs score	1.0 (1.0, 2.0)

Data are displayed as the median (interquartile range), or number (percentage)

*PR3-ANCA* Proteinase 3- antineutrophil cytoplasmic antibody, *MPO-ANCA* Myeloperoxidase-ANCA, *eGFR* estimated glomerular filtration rate, *TA/IF* renal tubular atrophy and interstitial fibrosis, *RRS* renal risk score, *BVAS* birmingham vasculitis activity score, *RVLs* renal vascular lesions

### Treatment and outcomes

Induction therapy included glucocorticoids in combination with MMF (*n* = 9), glucocorticoids in combination with CTX or rituximab (*n* = 51), or glucocorticoids alone (*n* = 34). 76 patients (80.9%) received intravenous methylprednisolone pulse therapy. In 22 patients (23.4%) plasma exchange therapy was performed. There was no statistically significant difference in the proportion of patients receiving different induction treatments between the RRS subgroups and the RVLs subgroups at baseline. The difference in the proportion of patients receiving different induction treatments to achieve nephrotic remission and ESRD between the RRS and RVLs subgroups was not statistically significant.

### Comparison of clinicopathologic characteristics

Groups were classified based on RRS, with 36 (38.3%), 43 (45.7%), and 15 (16.0%) patients falling into the low RRS, medium RRS, and high RRS groups, respectively. Similarly, the group of RVLs was divided into four groups: without RVLs, mild RVLs, moderate RVLs, and high RVLs with 13 (13.8%), 39 (41.5%), 29 (30.9%), and 13 (13.8%) patients, respectively. There were significant differences in SCr, eGFR, dialysis at diagnosis, SBP, percentage of normal glomeruli, and percentage of fibrous crescents among the RRS subgroups or the RVLs subgroups (*P* < 0.05) (Table 2). Clinical indicators in the high RRS group and severe RVLs group were significantly worse than those in the low RRS group and without RVLs group, respectively (*P* < 0.05). There were no significant differences among groups in ALB, CRP, C3, and C4.

**Table 2:** Evaluation of AAGN patients with RRS and RVLs score

	RRS Group			
	Low	Medium	High	*P*
SCr (μmol/L)	147.5 (108.8, 196.3)^b^	363.0 (247.0, 493.0)^ab^	673.0 (510.0, 780.0)^a^	< 0.001
eGFR (ml/min/1.73 m^2^)	41.9 (30.2, 64.4)^b^	12.2 (8.8, 17.5)^ab^	6.6 (5.3, 9.0)^a^	< 0.001
Dialysis at diagnosis (*n*, %)	0 (0)^b^	25 (58.1)^a^	13 (86.7)^a^	< 0.001
SBP (mmHg)	125 (120, 160)^b^	150(130, 180)^a^	151 (136, 180)^a^	0.004
Normal glomeruli (%)	58.0 (40.4, 79.2)^b^	20.0 (12.8, 38.5)^ab^	5.1 (2.7, 10.0)^a^	< 0.001
fibrocellular crescent (%)	0 (0, 10.0)^b^	10.0 (0, 26.8)^a^	5.9 (0, 16.0)^a^	0.011

	RVLs Group				
	Without	Mild	Moderate	Severe	*P*
SCr (μmol/L)	166.0 (118.0, 198.5)^b^	207.0 (132.0, 414.0)^b^	344.0 (182.0, 475.5)^ab^	532.0 (347.5, 752.0)^a^	< 0.001
eGFR (ml/min/1.73 m^2^)	37.8 (27.5, 48.0)^b^	25.5 (10.5, 42.0)^b^	13.8 (9.7, 26.9)^a^	8.0 (5.8, 13.6)^a^	< 0.001
Dialysis at diagnosis (*n*, %)	1 (7.7)^b^	11 (28.2)^b^	14 (48.3)^b^	12 (93.2)^a^	< 0.001
SBP (mmHg)	130 (123, 159)^b^	134 (120, 160)^b^	150 (128, 180)	180 (146, 188)^a^	0.004
Normal glomeruli (%)	60.0(39.3, 78.8)^b^	33.3 (16.7, 59.6)^ab^	20.0 (11.8, 52.5)^a^	12.5 (5.2, 31.7)^a^	0.004
fibrocellular crescent (%)	0 (0, 2.2)^b^	8.9 (0, 22.0)^a^	2.4 (0, 10.3)^b^	14.3 (6.5, 28.0)^a^	0.007

Data are displayed as the median (interquartile range)

*RRS* renal risk score, *RVLs* renal vascular lesions, *SCr* serum creatinine, *eGFR* estimated glomerular filtration rate, *SBP* systolic blood pressure

^a^Indicates *P* < 0.05 compared with the group with Low RRS or without RVLs

^b^Indicates *P* < 0.05 compared with the group with High RRS or severe RVLs

### Comparison of renal survival

**Fig. 2:**
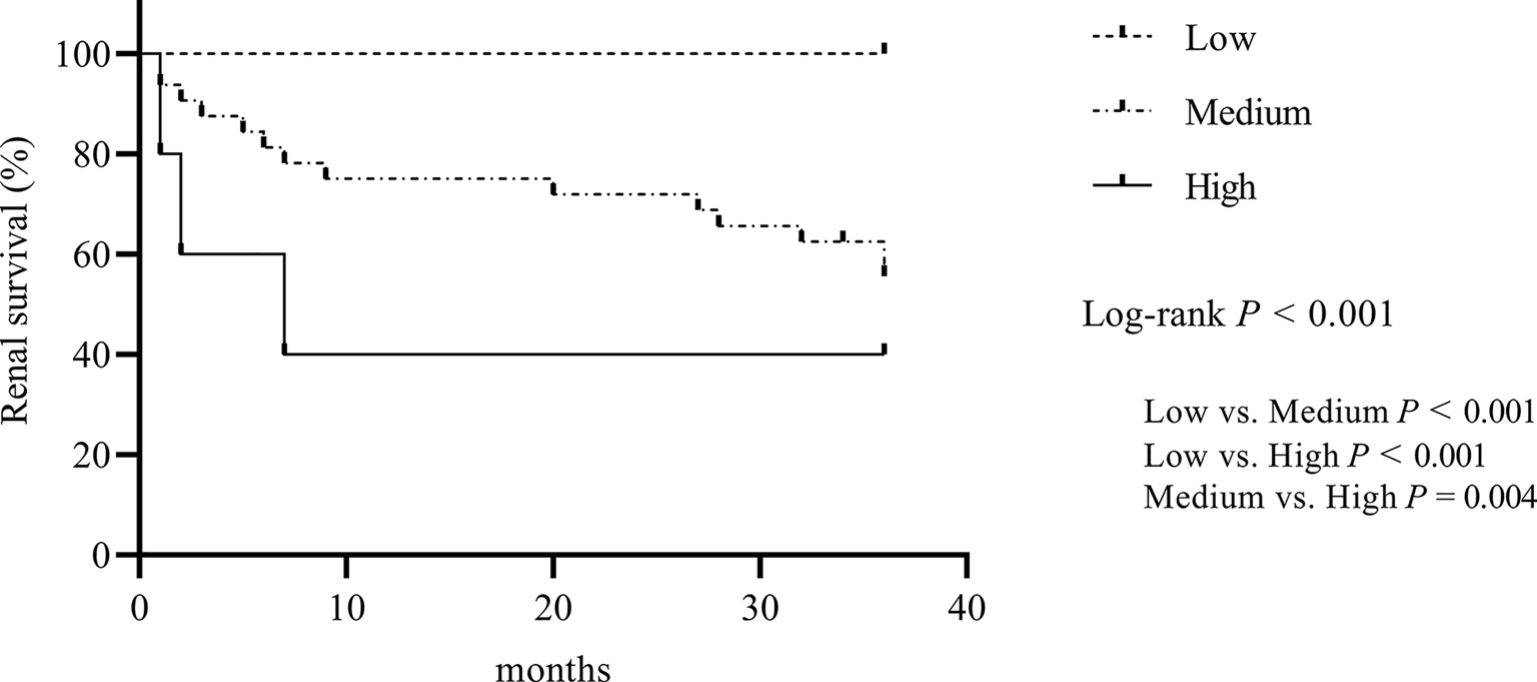
Kaplan–Meier curves demonstrating renal survival in the renal risk score

**Fig. 3:**
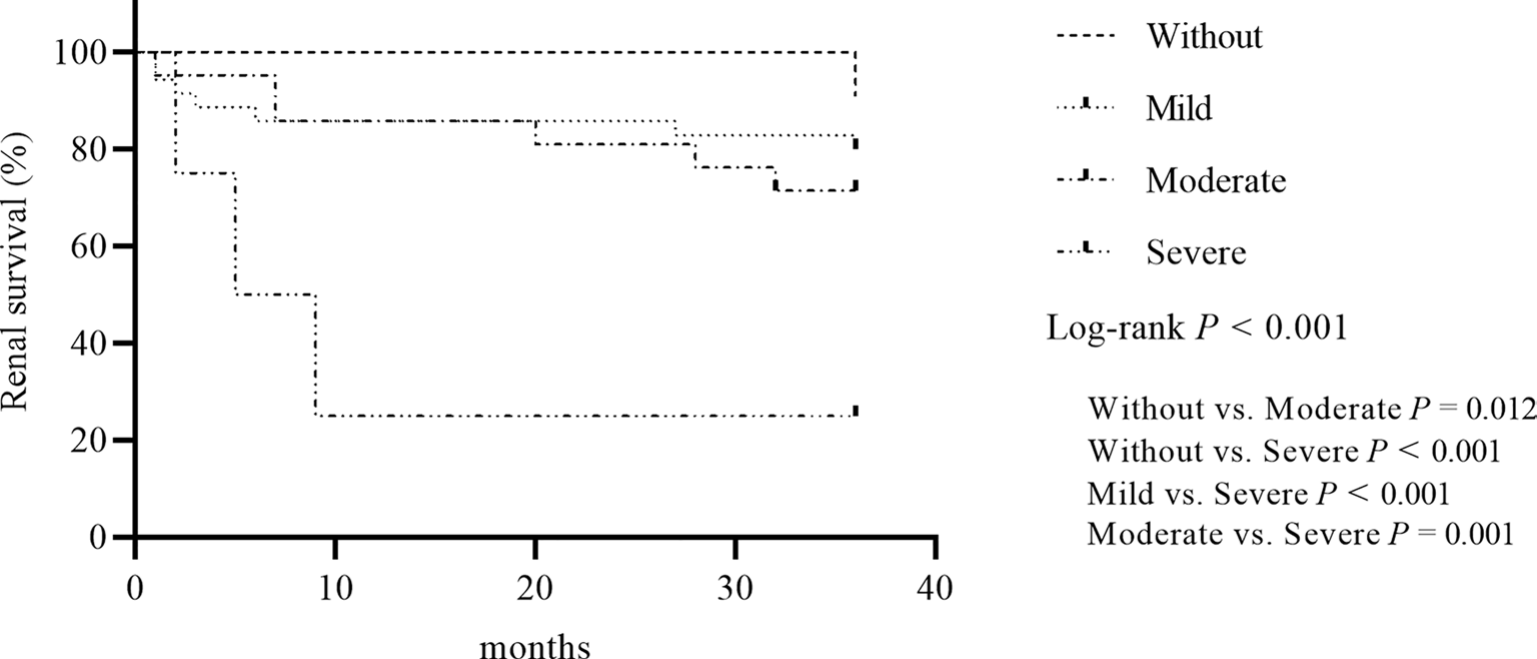
Kaplan–Meier curves demonstrating renal survival in the renal vascular lesions score

At the end of follow-up, the median follow-up time was 36 months, and 38 patients progressed to ESRD. The differences in renal survival were statistically significant among the three RRS subgroups (*P* < 0.001) (Fig. 2). Renal survival in the high RRS group was significantly worse than in the low RRS and medium RRS groups (*P* < 0.001, *P* = 0.004, respectively). Renal survival in the medium RRS group was significantly worse than in the low RRS group (*P* < 0.001). Significant differences in renal survival were statistically significant among the four RVLs subgroups (*P* < 0.001). Renal survival in the group with severe RVLs was significantly worse than in the group without RVLs, the group with mild RVLs, and the group with moderate RVLs (*P* < 0.001, *P* < 0.001, *P* = 0.001, respectively). Renal survival in the group with moderate RVLs was significantly worse than in the group without RVLs (*P* = 0.012). No difference in renal survival was observed between the group with mild RVLs and the group without RVLs (Fig. 3).

### Models of renal prognosis

The univariate Cox regression results showed that HB, UA, SCr, eGFR, SBP, urinary red blood cells, urinary protein, percentage of normal glomeruli, RRS, BVAS, and RVLs score were predictors of ESRD (*P* < 0.05) (Table 3). Multifactorial Cox regression excluding RRS, BVAS, and RVLs score, variables included in RRS and BVAS, showed that HB and UA were predictors of ESRD (*P* < 0.05). Combining age and sex to create the base model: age, sex, HB, UA. Logistic regression in MedCalc software was used to determine the composite predictor of RVLs score combined with RRS (RVLs score & RRS, RR) and the composite predictor of RVLs score, RRS combined with BVAS (RVLs score & RRS & BVAS, RRB). RRS, RVLs score, BVAS, RR, and RRB were added to the base model (Table 4). The results showed that the C statistic was significantly higher after the addition of RRS, RR, and RRB to the base model than before (*P* < 0.001, *P* < 0.001, *P* < 0.001, respectively). In addition, IDI and NRI were significantly increased by the addition of RRS, RR, and RRB.

**Table 3:** Analysis of factors influencing the occurrence of ESRD

Characteristics	Univariate cox regression			Multifactorial cox regression		
	*OR*	*95%CI*	*P*	*OR*	*95%CI*	*P*
Age	1.018	0.990–1.046	0.215	1.016	0.989–1.044	0.239
Gender	1.202	0.634–2.278	0.573	1.155	0.581–2.296	0.681
HB	0.967	0.947–0.987	**0.001**	0.969	0.949–0.989	0.003
UA	1.006	1.003–1.008	** < 0.001**	1.006	1.003–1.009	< 0.001
Scr	1.003	1.002–1.004	** < 0.001**			
eGFR	0.889	0.847–0.933	** < 0.001**			
SBP	1.016	1.005–1.028	**0.005**	1.009	0.996–1.022	0.197
urinary red blood cells	1.001	1.000–1.002	**0.018**			
Urine protein	1.227	1.077–1.399	**0.002**			
Normal glomeruli proportion	0.969	0.953–0.985	** < 0.001**			
tubular atrophy and interstitial fibrosis	1.995	1.473–2.701	** < 0.001**			
RRS	4.696	2.844–7.753	** < 0.001**			
BVAS	1.091	1.009–1.180	**0.030**			
RVLs score	2.557	1.717–3.808	** < 0.001**			

*HB* hemoglobin, * UA* uric acid, *SCr* serum creatinine, *eGFR* estimated glomerular filtration rate, *SBP* systolic blood pressure, *RRS* renal risk score, *BVAS* birmingham vasculitis activity score, *RVLs* renal vascular lesions

*P* < 0.05 indicates that the difference is statistically significant and is bolded in the table

**Table 4:** Prediction of renal prognosis by RRS, BVAS, RVLs score, RR, and RRB added to the base model

Characteristics	C statistic (95% *CI*)	*P*	IDI	Categorical NRI	Continuous NRI
Base model (reference)	0.796 (0.704–0.860)				
Base model plus RRS	0.883 (0.822–0.934)	** < 0.001**	0.172	0.204	0.677
Base model plus BVAS	0.812 (0.724–0.867)	0.234	0.022	0.098	0.374
Base model plus RVLs score	0.849 (0.777–0.897)	**0.06**	0.175	0.215	0.795
Base model plus RR	0.899 (0.842–0.943)	** < 0.001**	0.241	0.355	1.236
Base model plus RRB	0.918 (0.868–0.954)	** < 0.001**	0.286	0.445	1.169

*NRI* net reclassification improvement, * IDI* integrated discrimination improvement, *RRS* renal risk score, * BVAS* Birmingham vasculitis activity score, *RVLs* renal vascular lesions, *RR* RVLs score & RRS, *RRB* RVLs score & RRS & BVAS

*P* < 0.05 indicates that the difference is statistically significant and is bolded in the table

### Comparison of RRS, RVLs score, BVAS, and combined systems

**Fig. 4:**
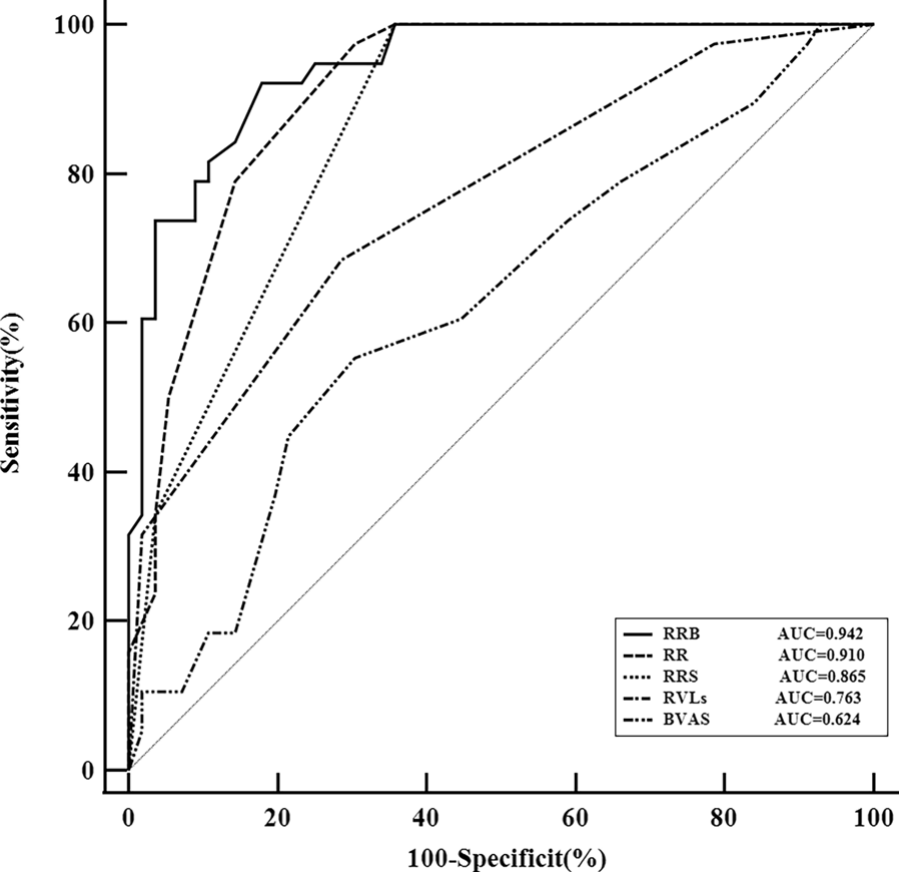
Comparison of ROC curves for RRS, RVLs score, BVAS, RR, and RRB. *ROC* receiver operating characteristic, *RRS* renal risk score, *RVLs* renal vascular lesions, *BVAS* Birmingham vasculitis activity score, *RR* RVLs score & RRS,* RRB* RVLs score & RRS & BVAS

In the sensitivity analysis, we evaluated the robustness of our results by comparing RRS, BVAS, RVLs score, RR, and RRB using receiver operator characteristic curve (ROC) (Table 5) (Fig. 4). The results showed that RRS, RVLs score, BVAS, RR, and RRB were statistically significant when used to predict renal prognosis. (*P* < 0.001,* P* < 0.001,* P* = 0.042, *P* < 0.001,* P* < 0.001, respectively). Further comparison showed that the AUC of RRB was significantly higher than that of RRS, BVAS, and RVLs score (*P* = 0.0011, *P* < 0.0001, *P* < 0.0001, respectively). There was no statistically significant difference in AUC between RRB and RR. The AUC of RR was significantly higher than that of RRS, BVAS, and RVLs score (*P* = 0.0164, *P* < 0.0001, *P* = 0.0001, respectively). The AUC of RRS was significantly higher than that of BVAS and RVLs score (*P* = 0.0006, *P* = 0.0463, respectively). There was no statistically significant difference in AUC between BVAS and RVLs score (*P* = 0.0752). The relationship between RRS, RVLs score, and RR was calculated using a linear fit and yielded the linear equation RR = -0.4766 + 0.1231 × RVLs score + 0.395 × RRS (*P* < 0.001).

**Table 5:** AUC of RRS, BVAS, RVLs score, and combined systems

Characteristics	AUC	95% *CI*	*P*
RRS	0.865	0.794–0.936	** < 0.001**
BVAS	0.624	0.508–0.740	**0.042**
RVLs score	0.763	0.664–0.861	** < 0.001**
RR	0.910	0.852–0.967	** < 0.001**
RRB	0.942	0.899–0.985	** < 0.001**

*AUC* area under the receiver operating characteristic curve, *RRS* renal risk score*, RVLs* renal vascular lesions, *BVAS* birmingham vasculitis activity score, *RR* rvls score and RRS RRB: RVLs score and RRS and BVAS

*P* < 0.05 indicates that the difference is statistically significant and is bolded in the table

### Renal survival was compared within the RR group

**Fig. 5:**
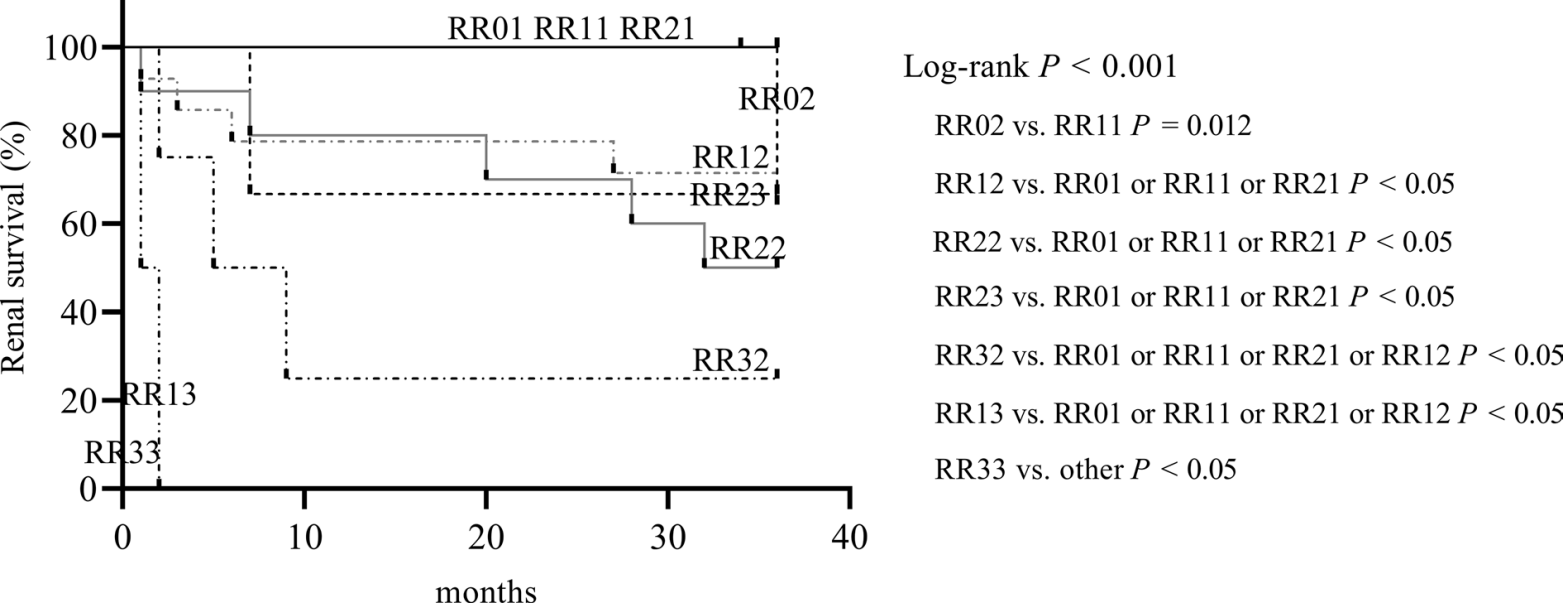
Kaplan–Meier curves demonstrating renal survival in the RR. *RVLs* renal vascular lesions, *RRS* renal risk score, *RR* RVLs score & RRS, *RR01* RVLs score 0&RRS 1(RR11, RR21, RR02, RR12, RR22, RR32, RR13, RR23, RR33 as above)

The Kaplan–Meier curve by RR classification showed significant differences in renal survival between groups (Log-rank *P* < 0.001) (Fig. 5). With the exception of RVLs score 2&RRS 3 (RR23) group, the higher the RVLs score at the same RRS level, the worse the renal prognosis. The RR33 group had a significantly worse prognosis than the other groups (*P* < 0.05). The renal prognosis of group RR13 or RR32 was significantly worse than that of groups RR01, RR11, RR21, and RR12 (*P* < 0.05). The renal prognosis of the RR23 group, RR22 group, or RR12 group was significantly worse than that of the RR01 group, RR11 group, and RR21 group (*P* < 0.05). The renal prognosis of the RR02 group was significantly worse than that of the RR11 group (*P* = 0.012).

## Discussion

The kidney is the most commonly affected organ in AAV, and despite intensive treatment, ESRD occurs in 20–25% of AAGN patients [9, 10]. At the same time, infections caused by excessive immunosuppression can increase short- and long-term mortality [11]. To guide diagnosis and treatment, prevent excessive immunosuppression, and reduce complications, it is urgent to find a comprehensive method for histopathological and clinical prediction of AAV.

We retrospectively collected clinicopathologic data from ninety-four patients with AAGN. In this study, up to 40% of patients progressed to ESRD, with 18% of ESRD patients dying during the study period. The higher incidence of ESRD and mortality may be related to the fact that our study center is a regional medical center with a high proportion of critically ill patients. Survival analysis revealed the best renal prognosis in the low RRS and without RVLs groups, and the worst renal prognosis in the high RRS and severe RVLs groups, suggesting that the RRS and RVLs score have high predictive power for renal outcome.

The RRS has been validated as a good prognostic tool for renal disease in Japan, Korea, Mexico, and Spain [5, 6, 12, 13]. In the present study, renal prognosis deteriorated sequentially in the low, medium, and high RRS groups, which is consistent with the findings of Brix et al. The present study has similar background characteristics to studies in Japan and Korea [14–16], but is in contradiction with their study, which showed a similar prognosis for the medium RRS group and the low RRS group [5, 6]. There are several possible reasons for this inconsistency. First, the duration of follow-up was different. The study in Japan had a longer follow-up period. After a longer follow-up period, the renal prognosis in the low RRS group and medium RRS group might be close to each other; second, the baseline level was different. In the Korean study, the proportion of normal glomeruli was lower (32.5% vs. 20.4%). In addition, the proportion of MPO-ANCA positive patients was higher (87.2% vs. 93.5%). Previous studies have shown that patients with MPO-ANCA have more severe histopathological renal changes than proteinase 3-ANCA positive patients [17]. More severe baseline indicators may indicate a higher risk of progression to ESRD.

The RVLs score is another scoring system for patients with AAGN that uses vascular lesions to predict renal prognosis. Mechanistically, when neutrophils are excessively activated by ANCA, abnormal cytokines are produced, which, together with the release of reactive oxygen species and lytic enzymes, lead to the excessive formation of neutrophil extracellular traps (NETs) and damage to vascular endothelial cells [18]. Our previous study showed that patients with RVLs have a worse survival prognosis than patients without RVLs [8]. On this basis, we found that RVLs was associated with renal prognosis, and the results showed that renal prognosis was worst in the group with severe RVLs. This finding supports the important role of vasculopathy in the progression of renal disease reported by Baradaran et al. [19]. The poor renal prognosis in exacerbation of vasculopathy may be due to microangiopathy caused by adaptive changes in hemodynamics during the early reduction of glomerular filtration rate in AAGN, which accelerates the loss of renal units and leads to further deterioration of kidney disease [20]. However, in the present study, renal prognosis was similar in the groups without RVLs and mild RVLs. We speculate that this may be related to the different response to treatment in the two groups of patients. In AAGN, renal tissue ischemia due to vasculopathy may cause angiotensin II -dependent TGF-β1 overexpression, which contributes to the development of renal fibrosis [21–23]. The relatively reversible renal injury in the mild RVLs group, which responded better to treatment compared with the moderate RVLs group, may reduce the prognostic difference between the without RVLs group and the mild RVLs group.

In addition to the within-group analysis, we compared the predictive power of RRS, BVAS, and RVLs score and showed that RRS and RVLs score were superior and noninferior to BVAS, respectively. The advantage of RRS is that it reflects both clinical laboratory parameters (eGFR) and histopathological parameters (normal glomeruli and TA /IF). Compared with clinical laboratory parameters alone (BVAS), this suggests that the proportion of normal glomeruli and tubulointerstitial damage are critical in predicting renal prognosis. Mechanistically, in AAGN, damaged tubular epithelial cells can cause acute and chronic inflammatory responses leading to interstitial inflammation and fibrosis, and the self-reinforcing cycle that forms between inflammations further exacerbates renal injury, suggesting that tubulointerstitial lesions are associated with poor renal prognosis [24, 25]. Conventionally, it is assumed that the percentage of normal glomeruli is the best predictor of renal prognosis and that a lower percentage of normal glomeruli may lead to a worse renal prognosis [26, 27].

To further improve the predictive power of renal prognosis, we compared a composite scoring system consisting of two or all three RRS, BVAS, and RVLs score. The results showed that the predictive power of RR and RRB was superior to that of RRS. The predictive power of RR and RRB was comparable. Considering that the multisystem and multipoint assessment of BVAS is prone to confounding and cumbersome [28, 29], we chose RR as the best predictor of renal prognosis in the present study. Finally, the relationship between RR subgroups and renal prognosis was analyzed using Kaplan–Meier curves. The results showed that, except for the RR23 group, the higher the RVLs score at the same RRS level, the worse the renal prognosis. The renal prognosis of the RR23 group should be the same as that of the RR13 group and worse than that of the RR02 group and the RR12 group, but the prognosis of the RR23 group was the same as that of the RR02 group and the RR12 group in this study. We suspect that this is due to the small sample size of the RR23 group, which biases the results of the present study. Nevertheless, the parameters of RR observation, including clinical indicators, glomerular, tubular, interstitial, and renal vessel-related pathological indicators, not only allowed the subdivision of subgroups and were easy to calculate but also were the best predictors of renal prognosis in this study.

There are the following limitations: (1) This is a single-centre study. Because of the low prevalence and poor prognosis of AAV, the sample size of this study was relatively small, which may have affected the power of the results. (2) This was a retrospective study, so there was limited documentation of the efficacy of different treatments on renal outcomes.

## Conclusion

In conclusion, in this study, the predictive power of RR for renal prognosis was better than that of RRS, BVAS, and RVLs score. It is suggested that the RR model should be used in clinical practice for the assessment of renal prognosis in patients with AAGN.

## Data Availability

The datasets used and analyzed during the current study are available from the corresponding author on reasonable request.

## Abbreviations

ANCA: Antineutrophil cytoplasmic antibody
AAV: ANCA-associated vasculitis
MPO: Myeloperoxidase
PR3: Proteinase 3
ESRD: End-stage renal disease
TA/IF: Rate of renal tubular atrophy and interstitial fibrosis
RRS: Renal risk score
BVAS: Birmingham vasculitis activity score
RVLs: Renal vascular lesions
SBP: Systolic blood pressure
DBP: Diastolic blood pressure
HB: Hemoglobin
ALB: Albumin
SCr: Serum creatinine
UA: Uric acid
eGFR: Estimated glomerular filtration rate
CRP: C-reactive protein
AUC: Area under the subject operating characteristic curve
RR: RVLs score and RRS
RRB: RVLs score and RRS and BVAS
